# Efficacy of the InvictDetect^TM^ Immunostrip^®^ to Taxonomically Identify the Red Imported Fire Ant, *Solenopsis invicta*, Using A Single Worker Ant

**DOI:** 10.3390/insects11010037

**Published:** 2020-01-01

**Authors:** Steven M. Valles, Charles A. Strong, Robert S. Emmitt, Christopher T. Culkin, Ronald D. Weeks

**Affiliations:** 1 Center for Medical, Agricultural and Veterinary Entomology, USDA-ARS, 1600 SW 23rd Drive, Gainesville, FL 32608, USA; chuck.strong@usda.gov; 2 Agdia, Inc., 52642 County Road 1, Elkhart, IN 46514, USA; Robert.emmitt@agdia.com (R.S.E.); chris.culkin@agdia.com (C.T.C.); 3 Animal and Plant Health Inspection Service, Plant Protection and Quarantine, Science and Technology, 920 Main Campus Drive, Suite 200, Raleigh, NC 27606, USA; ron.d.weeks@usda.gov

**Keywords:** biosecurity, immunological detection, invasive insect, lateral flow immunoassay

## Abstract

The early detection and identification of the red imported fire ant *Solenopsis invicta* are crucial to intercepting and preventing it from becoming established in new areas. Unfortunately, the visual identification of fire ants to species is difficult and ant samples must often be couriered to an expert for positive identification, which can delay control interventions. A lateral flow immunoassay that provides a rapid and portable method for the identification of *S. invicta* ants was developed and commercialized, and it is available from Agdia, Inc. under the trade name InvictDetect^TM^. While the test was 100% accurate when using the recommended minimum sample of three ant workers, InvictDetect^TM^ was field tested for the first time while using homogenates prepared from single *S. invicta* workers to determine the effectiveness of the method under these non-recommended conditions. Disregarding social form, the false negative rate was 25.5% for an initial single worker ant test and 10% after a repeat test was performed. The InvictDetect^TM^ false negative response was independent of worker weight. Though InvictDetect^TM^ requires a minimum of three worker ants, the test improves upon current identification methods because it can be conducted in the field, be completed in 10–30 min, and requires no special training or expertise.

## 1. Introduction

The red imported fire ant, *Solenopsis invicta* Buren (Hymenoptera: Formicidae), is a highly invasive ant species native to South America [1]. It has been listed as one of the most invasive species on the planet [2] and has been introduced into North America [3], the Caribbean [4], Australia [5], and throughout Asia [6,7]. *S. invicta* is currently considered one of the most damaging global insect pests [2]. The ant was introduced into the United States in the 1940s [8], where it is estimated to cost $6 billion annually to control and repair the damage it causes [9]. In areas where the ant is established and in potentially new areas of introduction, the rapid detection of the ant is essential to its interception and the prevention of its additional range expansion and/or establishment in new areas. The positive taxonomic identification of intercepted ant species invariably relies on an ant expert. Unfortunately, shipping specimens to a qualified taxonomist is often necessary and delays their identification. A method that is capable of being used at the point-of-need to identify *S. invicta* would greatly facilitate identification and timely intervention.

In response to this need, a rapid, field-capable assay was developed to discriminate *S. invicta* from all other ant species [10]. The method targets the *Solenopsis invicta* venom protein 2 (Soli2), which is unique to fire ants [11]. The test provides the rapid identification of *S. invicta* ants in 10–30 min, requires no special equipment, and can be completed by untrained personnel in the field.

Monoclonal antibodies were designed against two regions of the Soli2 venom, and a sandwich lateral flow immunoassay (LFA) was developed. One antibody captures the Soli2 venom protein from an ant homogenate, and the other, at a different position on the venom protein, reports its presence. The LFA was found to be species-specific against U.S. and Australian species of ants [10,12]. The technology was later commercialized as InvictDetect^TM^ Immunostrip^®^ (Agdia, Elkhart, IN) and is now available for purchase by the public (https://orders.agdia.com/invictdetect-isk-49700-0010). The original LFA [8] required three-to-five worker ants for analysis, a number that is also recommended for the InvictDetect^TM^ Immunostrip^®^. While the discovery of a single ant is highly unlikely, investigators have criticized the requirement for three-to-five worker ants for the InvictDetect^TM^ Immunostrip^®^ test [13,14]. The objective of this research addresses this concern by examining the efficacy of the InvictDetect^TM^ Immunostrip^®^ when interrogating the homogenate of a single worker ant in the field.

## 2. Materials and Methods 

### 2.1. InvictDetectTM Field Tests

Field evaluations were conducted in and around Gainesville, Florida. Worker ants were collected from *S. invicta* nests by plunging a 20 mL glass scintillation vial, with the inner rim coated with talcum powder, into their raised dirt nest. Worker ants climbed the outside and fell into the vial, where they were unable to escape. Once a minimum of 30 ant workers had been collected, one worker ant was selected at random and tested with an InvictDetect^TM^ Immunostrip^®^. If a false negative response was indicated after 30 min, the test was repeated, again using a single worker chosen at random. The hypothesis being tested was simply that false negative responses do not occur when using a single *S. invicta* ant worker with the InvictDetect^TM^ Immunostrip^®^. The total number of nests tested were 52 (monogyne) and 18 (polygyne). The remaining sample of workers was returned to the laboratory and examined under a dissecting microscope to confirm that they were *S. invicta* [15]. In addition, the social form (polygyne or monogyne) was determined by PCR amplification of the *Gp-9* alleles, as described by Valles and Porter [16]. The proportion of nests that returned a false negative response for the initial and repeated tests was scored for each social form. 

### 2.2. Influence of Worker Weight on InvictDetectTM Response

*S. invicta* is polymorphic; workers exhibit a range of sizes associated with division of labor [17]. An experiment was conducted to determine the relationship between worker size (weight) and the false negative response rate of InvictDetect^TM^ Immunostrips^®^ by using a single ant worker. Six *S. invicta* colony samples were obtained by the scintillation vial method of collection, as described above, and immediately returned to the laboratory. Worker ants from each colony sample were selected at random and weighed. The sample size of workers weighed from each colony was 3, 4, 6, 10, 10, and 10 (monogyne colony samples; overall *n* = 43) and 3, 6, 7, 7, 10, and 10 (polygyne colony samples; overall *n* = 43). The individual worker ants were immediately homogenized in 100 μL of an elution buffer provided with the Agdia, Inc. detection kit, and the homogenate was tested with an InvictDetect^TM^ Immunostrip^®^. After 30 min, the response was scored. Positive and false negative responses were plotted against individual worker weight. The false negative response rate between the monogyne and polygyne colonies (disregarding weight) was compared with a Student’s *t*-test [18]. A Student’s *t*-test was also performed to compare the mean worker weight from the polygyne and monogyne colonies rendering a false negative or positive response. To address the possibility of an effect caused by different nests, the responses were compared by an analysis of variance (General Linear Model) that used “nest” as a classifier.

## 3. Results

**Figure 1:**
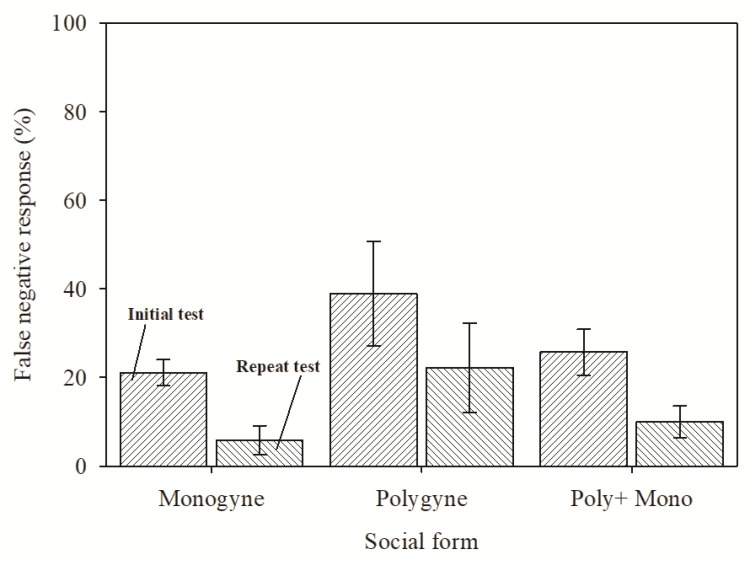
Mean (± standard error) proportion of InvictDetect^TM^ test strips yielding a false negative response when using a single ant worker from monogyne and polygyne *Solenopsis invicta* fire ant colonies in the field. Ants were removed from nests, and the immunoassay was conducted in the field. The “poly + mono” grouping summarizes data from both social forms. The left bar for each group represents the immunoassay results from the initial worker ant, and the right bar represents the results from the second worker ant (repeated assay). To address the possibility of an effect caused by different nests, the responses were compared by an analysis of variance that used “nest” as a classifier. “Nest” did not have a significant effect on the false negative response rate in either monogyne (F = 0.76; df = 10, 41; *p* = 0.67) or polygyne (F = 0.28; df = 5, 12; *p* = 0.92) ants.

Against individual worker ants from the monogyne colonies, InvictDetect^TM^ yielded a false negative response rate of 21.1% (Figure 1). Repeating the assay with a second individual worker reduced the false negative response rate to 5.8%. The false negative response rates for individual workers from the polygyne colonies were 38.9% for the initial test and 22.2% for the repeated test. When disregarding the social form and combining the data, a 25.7% false negative rate was observed for the first individual worker ant and 10% after the repeated test. For comparison, five pooled workers evaluated by InvictDetect^TM^ yielded a 100% positive response (i.e., no false negative responses; data not shown).

**Figure 2:**
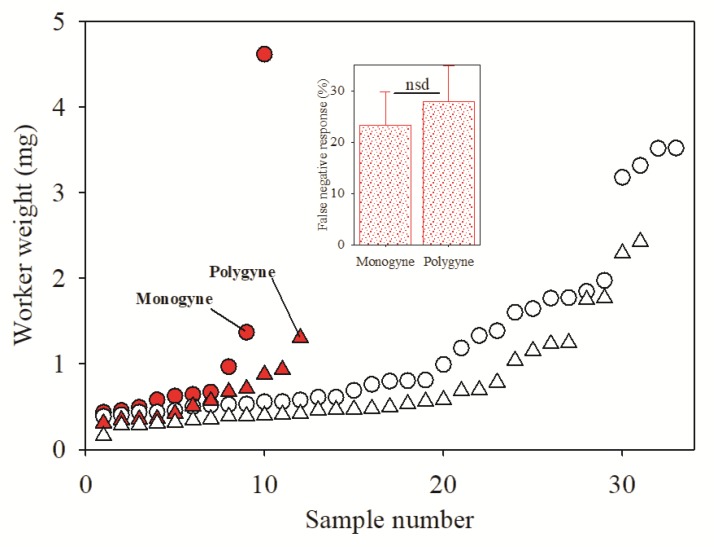
Influence of worker ant weight on the response of the InvictDetect^TM^ Immunostrip^®^. Each symbol represents the response from an individual worker ant from a monogyne (n = 43) or polygyne (n = 43) colony. Open symbols represent positive InvictDetect ^TM^ responses, and red symbols represent false negative responses; triangles = polygyne, and circles = monogyne. Inset: Mean (± standard error) false negative responses from monogyne and polygyne individual workers. NSD = no significant difference by Student’s *t*-test (t = 0.49; df = 84; *p* = 0.63).

Experiments where individual ants from both social forms were weighed and evaluated by InvictDetect^TM^ revealed that the false negative response was independent of the social form and weight of the individual worker ant used in the test (Figure 2). The mean weight of the individual workers from the monogyne (1.18 ± 1.02 mg) colonies was significantly (t = 2.7; df = 84; *p* = 0.009) heavier than worker ants sampled from the polygyne colonies (0.71 ± 0.53 mg). However, there was no significant difference (t = 0.49; df = 84; *p* = 0.63) observed in the false negative response rate for individual workers between the monogyne (23.3 ± 6.5%) and polygyne (27.9 ± 6.9%) colonies (Figure 2, inset). 

**Figure 3:**
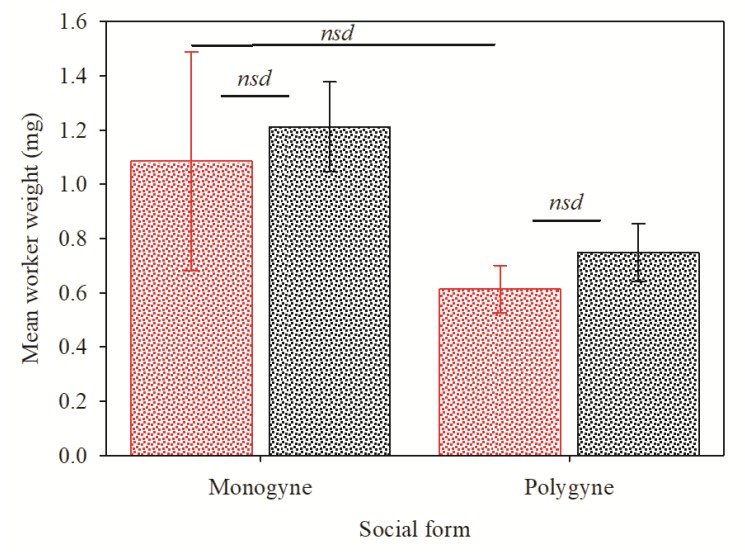
Mean (± standard error) worker weight of monogyne and polygyne worker ants that produced a positive and false negative InvictDetect ^TM^ Immunostrip ^®^ response. Red bar on the left represents the false negative response, and black bar on right represents the positive response. NSD = no significant difference by Student’s *t*-test for monogyne false negative versus positive response (t = −0.34; df = 41; *p* = 0.74); polygyne false negative versus positive response (t = −0.98; df = 41; *p* = 0.34); and false negative response between monogyne and polygyne ants (t = 1.15; df = 20; *p* = 0.28).

**Figure 4:**
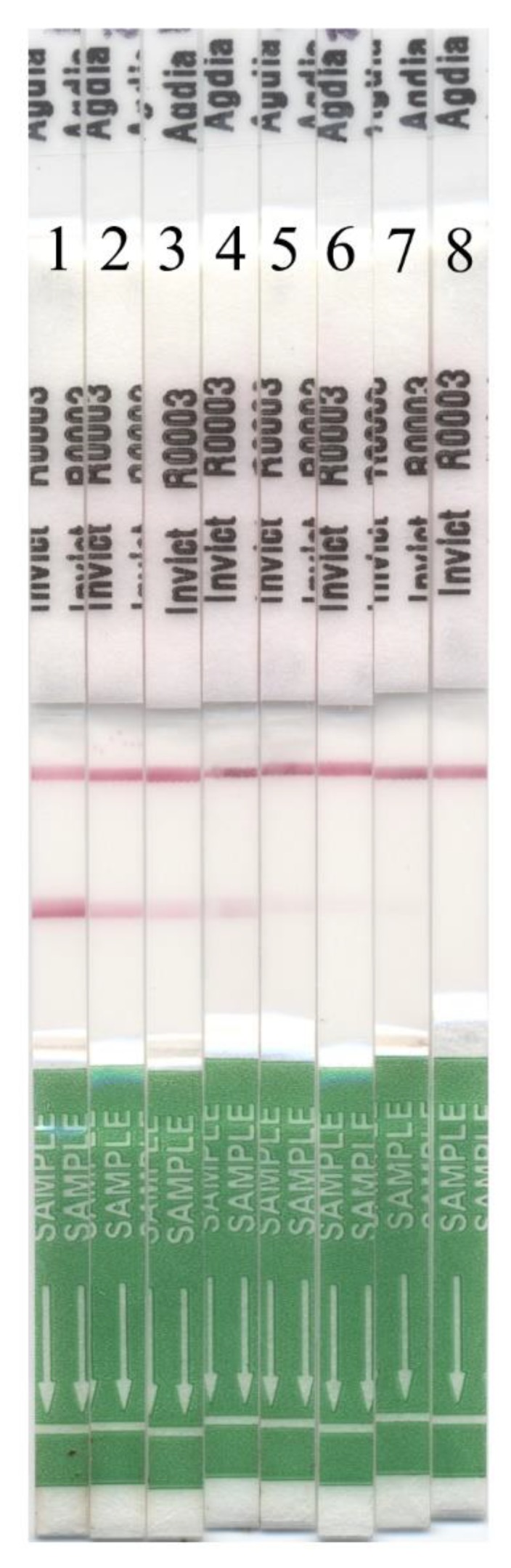
Range of responses from InvictDetect^TM^ Immunostrips^®^ interrogated with a single worker ant. Samples 1–7 are positive responses. Sample 8 is a negative control.

The mean weight of the polygyne workers that produced a false negative (0.61 ± 0.30 mg) was not significantly different (t = 1.15; df = 20; *p* = 0.28) from the mean weight of the monogyne workers that produced a false negative response (1.09 ± 1.27 mg) (Figure 3). Furthermore, the mean weight of the individual worker ants that produced a false negative response was not significantly different from the weight of the individual ants that produced a positive InvictDetect^TM^ response for the monogyne (t = −0.34; df = 41; *p* = 0.74) and polygyne (t = −0.98; df = 41; *p* = 0.34) social forms (Figure 3). Among tests with individual worker ants, the InvictDetect^TM^ response intensity was varied (Figure 4) and occasionally required a full 30 min incubation time before a response was clearly observable.

## 4. Discussion

Early detection and identification are crucial to intercepting *S. invicta*, initiating response measures, and preventing it from becoming established in new areas. Unfortunately, the visual and molecular identification of fire ants, including *S. invicta*, to species is difficult. Ant samples often must be couriered to an expert for positive identification, which can delay control options. In response to this need, a lateral flow immunoassay that provides a rapid and portable method for the identification of *S. invicta* ants was developed [10]. The *Solenopsis invicta* lateral flow immunoassay provides a new tool for the early detection and identification of *S. invicta* in the United States and abroad to facilitate interceptions, speed up identification and confirmation processes, and help to limit the spread of this invasive ant. The method was commercialized in 2017 and is now available on the open market from Agdia, Inc. under the trade name InvictDetect. The InvictDetect^TM^ Immunostrip^®^ provides a method that can be used in the field without any special equipment or training. Ants are collected and homogenized in the extraction buffer provided for 1 min. An InvictDetect^TM^ Immunostrip^®^ is placed in the ant homogenate, and a response is rendered within 10–30 min. The antibodies target the Soli2 venom protein. The quantity of Soli2 has been reported to vary with the age and size of the worker [19], so InvictDetect^TM^ requires three-to-five worker ants to ensure that a sufficient quantity of the venom protein is present for correct identification [10]. The requirement of three to five ants for the InvictDetect^TM^ test was recently criticized by Kim et al. [13] and Nakajima et al. [14], who suggested that during early stages of *S. invicta* invasions, the number of individual workers intercepted could be less than three. Thus, we field tested InvictDetect^TM^ Immunostrips^®^ against homogenates prepared from single *S. invicta* workers to determine the effectiveness of the method under these conditions. The false negative rate observed for monogyne and polygyne individual workers was 21.1% and 38.9%, respectively. The false negative rate was decreased to 5.8% (monogyne) and 22.2% (polygyne) when a repeat test was performed, again using a single ant worker each (i.e., two determinations with a single ant worker). Disregarding the social form, the false negative rate was 25.7% for the initial single worker ant test and 10% after a repeat test was performed. Thus, when two individual ant workers from a colony were interrogated separately with InvictDetect^TM^ Immunostrips^®^, positive identification was 90%. Therefore, if multiple workers are present, it is more logical to use a pooled group (three-to-five) where the test renders 100% accuracy.

Because *S. invicta* is a polymorphic species [17], we were curious to know if the InvictDetect^TM^ false negative responses when using a single worker ant were related to the mass/size of the ant worker. If such a relationship existed, it might be possible to improve the efficacy of InvictDetect^TM^ with a single worker ant by selecting the largest workers. However, our tests indicated that the InvictDetect^TM^ false negative response was independent of worker weight. Figure 2 illustrates that false negative responses were observed in workers weighing as much 4.62 mg (monogyne) and that positive responses occurred in the lightest workers (0.16 mg; polygyne). Furthermore, there were no significant effects of mean worker ant weight on false negative or positive responses (Figure 3). Indeed, even though worker ants in polygyne colonies are significantly smaller than in monogyne colonies [20], the InvictDetect^TM^ false negative response rate was not statistically different between the two social forms. Several studies have reported strong relationships between venom quantity and worker size [19,21,22]. In all of these studies, venom quantity was determined by either measuring the volume of the venom sac or the quantity of alkaloids it contained; none examined the production of venom proteins, such as Soli2. Our experiments confirm these reported variations in venom because the InvictDetect^TM^ tests that used individual worker ants had a false negative rate of about 25%. While this failure rate precludes the effective use of individual workers with the InvictDetect^TM^ Immunostrip^®^, we argue that detection of a single ant is an unrealistic scenario. Considering the social nature of *S. invicta*, it is more likely that a trail of foraging ants or an entire nest would be detected, thus permitting the collection of the needed three to five workers for evaluation with InvictDetect^TM^.

When the recommended number of worker ants (i.e., three to five) is used, the false negative rate of InvictDetect^TM^ is less than 1% with a single determination [10]. While there are PCR-based tests available [13,14] with greater sensitivities for detecting *S. invicta* than InvictDetect^TM^, they require a nucleic acid purification step, multiple reagents, long preparation and analysis times, specialized equipment, specialized training, and are not field portable. 

## 5. Conclusions

InvictDetect^TM^ is a commercially available lateral flow immunoassay field test that is used to identify *Solenopsis invicta* ant workers. Field tests were conducted to determine if the kit was able to discriminate *S. invicta* workers when using a single worker ant sample. Under these conditions and disregarding social form, a false negative test rate of 25.7% was observed when using a single ant worker. Despite exhibiting size polymorphism, the InvictDetect^TM^ response was independent of *S. invicta* worker weight. Because the threshold for detection of this invasive species is critical, a single worker ant is insufficient when using InvictDetect^TM^. However, a 100% positive response with the recommended three to five worker ants confirms that InvictDetect^TM^ is an important biosecurity tool for intercepting *S. invicta*.
